# An applied paradigm for simple analysis of the lower limb kinematic chain in explosive movements: an example using the fencing foil attacking lunge

**DOI:** 10.1080/23335432.2018.1454342

**Published:** 2018-03-25

**Authors:** Francis Mulloy, David R. Mullineaux, Phillip Graham-Smith, Gareth Irwin

**Affiliations:** a School of Sport and Exercise Science, University of Lincoln, Lincoln, UK; b Sport Science Department, Aspire Academy, Doha, Qatar; c Cardiff School of Sport, Cardiff Metropolitan University, Cardiff, UK

**Keywords:** Biomechanics, angular, velocity, proximal, distal, kinetics, sport

## Abstract

A simple method to quantify the kinematic chain in a propulsive task would facilitate assessment of athlete effectiveness. The study’s aim was to assess if the kinematic chain distinguishes between skill levels. Fencers were separated into two groups based on attacking lunge ability (7 skilled; 8 novices). Rear leg 3D joint angular extension velocity magnitudes and timings, sword kinematics and rear leg kinetics were obtained in the propulsion phase of the attacking lunge. Skilled fencers obtained greater sword velocity (3.24 ± 0.24 m∙s−1 vs. 2.69 ± 0.29 m∙s−1; *p* = 0.02). The skilled group had a greater sequential kinematic chain of the hip, knee and ankle, demonstrated by significantly greater ankle angular velocity (9.1 ± 2.1 rad·s−1 skilled; 5.4 ± 2.9 rad·s−1 novice).  Ankle plantarflexion velocity showed a strong positive correlation with horizontal peak force (*r* = 0.81; *p* < 0.01). The skilled group demonstrated greater horizontal impulse (1.85 ± 0.29 N·s·kg−1 skilled; 1.45 ± 0.32 N·s·kg−1 novice), suggesting greater effectiveness in applying the kinematic chain towards horizontal propulsion. Analysis of the kinematic chain, which was able to distinguish between skill levels in a propulsive task, is an effective and simple paradigm to assess whole limb contributions to propulsive movements.

## Introduction

The human musculoskeletal system predominantly translates joint rotations into linear movement, which requires the coordination of many skeletal muscles around multiple joints. Successful movement is achieved through an optimal kinematic sequence of these joints. According to Bunn’s (1972) ‘summation of speed principle’ kinematic sequencing augments an accumulation of angular velocities, generated in preceding segments, toward a distal end point. This has been demonstrated in a number of open-chain movements where the most distal segment is unattached, thus free to move. For example, ball kicking (Putnam 1991; Katis et al. 2015) handball throwing (Wagner et al. 2012), the tennis forehand (Landlinger et al. 2010), and the golf swing (Zheng et al. 2008; Tinmark et al. 2010).

In ‘closed chain’ movements, such as in jumping, the distal endpoint is fixed. The movement requirements therefore may not be maximal segment end point velocity, but rather whole body propulsion. In propulsive movements, such as jumping, the lower limb has also been shown to resemble a stereotypical proximal to distal sequence prior to take off (Bobbert and van Soest 2001). This sequential action seemingly contradicts mechanical optimization principles, where simultaneous extension of the hip, knee, and ankle plantar flexion is suggested as optimal (Gregoire et al. 1984). The neuromuscular anatomy of the lower limb, however, allows a proximodistal sequence to capitalize on the role of bi-articular muscles, allowing a transfer of energy between joints (Gregoire et al. 1984; Bobbert and van Soest 2001; Cleather et al. 2015). Muscular modelling has demonstrated that bi-articular muscles allow a net transfer of power between joints. For example, the rectus femoris has been calculated to transfer 21% of the relative work done in knee extension from the hip in single leg jumping, and 31% in sprinting (Jacobs et al. 1996). This relationship also exists with the gastrocnemius coupling the knee and ankle joints in plantarflexion. Mathematical modelling demonstrates the effectiveness of this lower limb rigid body chain in turning joint segment angular velocity into linear centre of mass velocity (Bobbert and van Soest 2001), making the sequential kinematic chain applicable to many propulsive sporting skills.

Frequently in applied sports biomechanics, individual joints are singled out as determinants of performance (e.g. rear knee extension in fencing; Bottoms et al. 2013). With the inherent complexity of joint interaction within coordinated human movement this single joint approach may be viewed as too simplistic. Approaches applying mathematical and muscular modelling (as above) or coordination paradigms (e.g. vector coding or continuous relative phase analysis) may combat this; however, these may be overly complicated for use in applied settings. Quantifying the whole lower limb by overlaying joint angular velocities could be a simple and useful method. Simple visualization could help a coach to identify athlete effectiveness in capitalizing on whole lower limb coordination. This would allow a coach or biomechanist to assess whether an increased contribution in one joint has consequences on a distal joint due to segmental interaction (e.g. overcoming additional inertia). Such an approach could provide a paradigm that differentiates between athletic ability in harnessing a task specific strategy, such as forward propulsion of the body’s centre of mass.

To investigate simple quantification of the lower limb kinematic chain in a sport specific skill, a movement needs to be selected. The fencing attacking lunge is appropriate, with the objective being to generate maximal forward propulsion, covering ground quickly to strike an opponent with a sword (Guilhem et al. 2014). Significantly, greater sword velocity in elite fencers has been attributed to more than arm extension velocity alone, with sword arm movement in coordination with the lower limb lunge distinguishing skilled from novice fencers (Yiou and Do 2000). Research has demonstrated that more skilled fencers coordinate timing of the lead kick out foot more effectively, with a delay between sword movement and foot movement (Gutiérrez-Dávila et al. 2013), however this has not explained where additional propulsion, underpinning sword velocity, is generated. Rear knee range of motion and peak rear hip flexion have been identified as significant predictors of sword velocity (Bottoms et al. 2013), however the relationship between the two has not been explored further. Kinematic and electromyographical data of elite fencers supports that the rear leg extensor muscles activate mainly in the propulsive phase of the attacking lunge (Guilhem et al. 2014), with this activation suggesting a temporal sequence in the rear leg with more distal muscles, such as plantar flexors in the ankle, firing later. These results allude to specific kinematic sequencing and suggest that skilled fencers harness a sequential kinematic chain to attain greater forward velocity. Therefore, this skill has been identified as a vehicle to investigate the use of the rear leg kinematic chain as an indicator of skill proficiency.

The aim of this research was to: (a) identify differences in the kinematic chain associated with skill level, using the fencing attacking lunge as an example, and (b) empirically demonstrate how these differences relate to a performance output such as forward sword velocity. It was hypothesized that skilled participants, in comparison to lesser skilled participants, would demonstrate (a) clearer proximal to distal sequencing of the rear leg kinematic chain through increasing angular velocity magnitudes at the hip, knee and ankle joints, and (b) greater accumulative angular velocity magnitudes.

## Methods

### Participants

Fifteen participants (mean ± SD; 8 novice; 6 M and 2 F, age 22 ± 10 years, height 1.74 ± 0.09 m, leg length 0.89 ± 0.06 m, mass 74.6 ± 16.2 kg, and; 7 skilled 5 M and 1 F; age 24 ± 14 years, height 1.78 ± 0.07 m, leg length 0.95 ± 0.08 m, mass 72.0 ± 15.3 kg) agreed to take part in this study and provided informed consent. Group anthropometrics, as outlined above, were not significantly different (independent *t*-test, *p* > 0.05). All participants had a minimum of one year of experience fencing with a foil weapon (skilled 8.2 ± 7.6 years, novice 6.9 ± 12.2 years), however to be classified as skilled participants had to be competitive at regional level or above, with three having competed nationally and two internationally. Additional inclusion criteria for skilled grouping maintained that individuals were capable of lunging over one leg length in distance and could achieve a sword velocity of over 3 m∙s^–1^ (Yiou and Do 2000).

### Procedure

Data collection took place at two institutions, with each participant visiting one of the two sites on one occasion. The multisite data collection was adopted to increase sample size. To avoid discrepancies between testing protocols, the same researcher conducted all data collection sessions at both sites.

**Figure 1.**
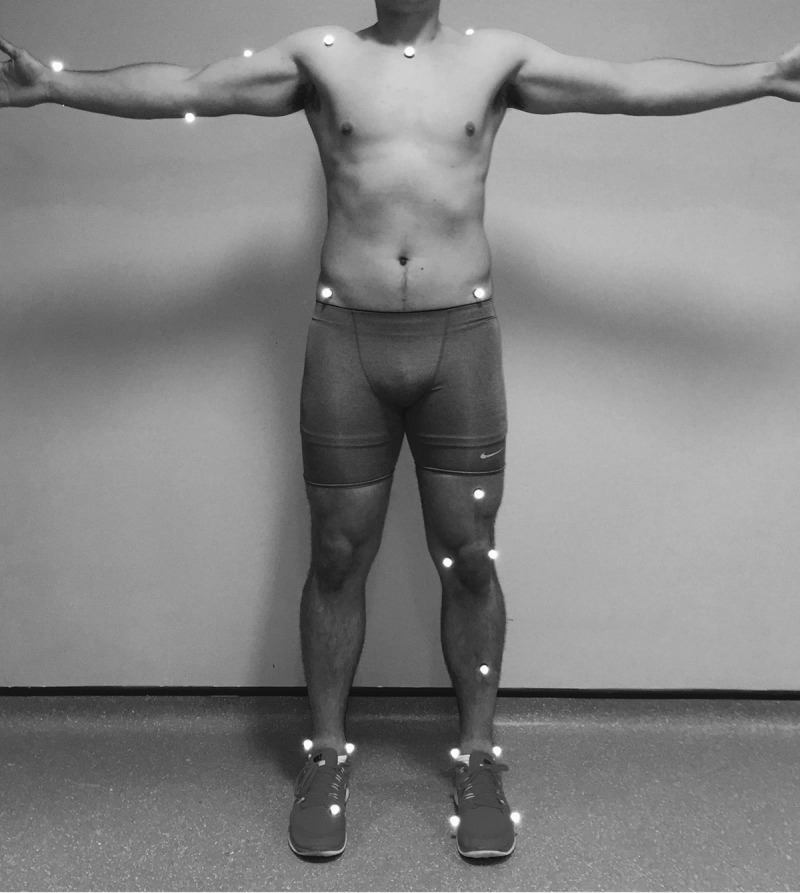
Marker placement for participants

All procedures were approved by the institutes’ ethics committee. At each site, testing took place in one day with participants completing seven lunges toward a 15 × 15 cm square target marked on the chest of the local fencing coach, with the top of the target individually set at the height of the participant’s sternal notch. Each participant wore tight fitting shorts (with females wearing a short vest top), along with their normal fencing shoes and competition foil. A total of 27 passive retro-reflective markers of 12.5 mm diameter were placed on the participant in anatomical landmarks for the rear leg, lead foot, pelvis, trunk and sword arm (see Figure 1 for specific marker locations). Anatomical bony landmarks were used over segmental clusters to ensure the approach used was accessible to applied practitioners (e.g. for video digitization). An additional three markers were placed on the sword (5 cm distal from the base, middle of the blade and 5 cm from the tip) and four on the target.

Rear leg markers: 1st and 5th metatarsal, lateral and medial malleolus, lateral and medial femoral epicondyle, and greater trochanter; lead leg: 1st metatarsal, and lateral and medial malleolus; pelvis markers: both Anterior and Posterior Superior Iliac Spines; trunk: both acromion processes and sternal notch; and sword arm: anterior and posterior shoulder, lateral and medial humeral epicondyle, and lateral and medial styloid processes. Additionally, single segmental markers were also placed on the shank, thigh, upper arm and lower arm for segment identification in the motion capture software.

The target centre was marked for participants to direct their lunges. Participants stood a self-selected distance (2.16 ± 0.07 m skilled and 2.01 ± 0.03 m novice; independent *t*-test *p* < 0.05) from the target, deemed their competitive attacking distance. With both feet on individual force plates, the participants were then requested to drop into the ‘on guard’ position and instructed to propel themselves forward as quickly as possible to strike the target centre upon reacting to an auditory signal. Only successful trials where the participant struck the target were analysed. Marker tracking was completed at site one using 12 cameras operated through Cortex v5.0.2 software (Motion Analysis Corporation, Santa Rosa, CA), and at site two using 16 cameras operated through Vicon Nexus v2.0 (Vicon Motion Systems ltd., Oxford, UK). Differences in kinematic collection setup were as a result of available resources at the respective institutions. Kinematic data at 200 Hz were synchronized at both sites with two Kistler force plates (Kistler type 9284, Kistler AG, Winterthur, Switzerland) via a standard analogue to digital synchronization cable, and sampled at 1000 Hz. The *Y* axis of the global coordinate system was orientated from the start position to the target, with the *Z* axis defined in the vertical direction and the *X* axis as the cross product of *Y* and *Z*.

### Data processing

A custom written Matlab code (R2015a, Mathworks, Natick, MA) was used to analyse each trial. All data were smoothed using a zero lag, fourth-order, low pass Butterworth filter at 10 Hz for kinematic and 50 Hz for kinetic data. Filter cut-off frequencies were selected based on previous fencing research for the kinematics (Guilhem et al. 2014), and visual inspection showed that 50 Hz allowed for the removal of electrical noise without affecting the kinetic data peaks. Kinetic data from the rear foot force plate were extracted and interpolated to 101 data points to allow comparison with kinematic data. Two key events were identified using Matlab; onset of force (*F*_*O*_) and take off (*F*_*TO*_). *F*_*O*_ was defined as the first instance the rear leg resultant force vector (*F*_*R*_) > 20 N of initial force, and *F*_*TO*_ as the first instance *F*_*R*_ < 50 N. Push off (*F*_PushOff_) was the phase defined from *F*_*O*_ to *F*_*TO*_ and time normalized from 0 to 100% respectively. Prior to the calculation of impulse body weight was removed from vertical ground reaction force (*F*_*Z*_), and the mean of the first 10 frames of the horizontal ground reaction force (*F*_*Y*_) used to offset to zero. The integral of both *F*_*Z*_ and *F*_*Y*_ were obtained using the trapezoidal method to calculate net vertical and horizontal impulse (Impulse_*Z*_ and Impulse_*Y*_ respectively). Kinetic variables were normalized to body weight to allow for comparison between participants.

Each whole lunge movement was analysed from the onset of sword movement (defined as horizontal sword velocity >0.2 m.s^−1^), up until target contact. A virtual target centre was calculated as the mean of the four target markers. Target contact was deemed as the time instant at which peak acceleration of the target centre occurred as a consequence of sword impact. Total movement time was determined from first sword movement until target contact. Forward horizontal sword velocity was obtained from the most proximal sword marker. The rear hip joint centre was calculated from relative Anterior Superior Iliac Spine breadth (14% medial, 19% posterior and 30% distal; Bell 1990). The sword arm shoulder virtual joint centre was calculated as the midpoint between anterior and posterior markers placed at estimated joint centers and directly posterior to the acromion process, while the shoulder was abducted to 90°. Four further virtual joint centers were calculated for the sword arm wrist and elbow, as well as rear knee, rear ankle and lead ankle using standard medial and lateral bony landmark markers. Lunge distance was determined as maximal forward displacement of the front foot virtual ankle joint centre in the foot-target plane, and normalized to leg length (vertical height of rear leg greater trochanter marker).

Initial pilot testing demonstrated that the lead foot left the ground in a kick out action almost at the initiation of the movement, thus contributed little to the propulsive ground reaction force. In addition, lead leg joint kinematics were highly variable between and within individuals, therefore the front leg was omitted from the analysis.

Three dimensional joint angles and angular velocities were calculated as vectors from virtual joint centers. Hip angle was calculated as the angle between two vectors running from the virtual hip to the virtual shoulder and from the virtual hip to virtual knee respectively. Extension and plantar flexion was deemed as positive, with full extension defined as 180° for all joints. Series kinematic data were interpolated to 101 data points from *F*_*O*_ to *F*_*TO*_, allowing presentation of angular velocities as a percentage of the push off phase with an additional 25 points for the early flight phase for visual clarity. Temporal variables were identified as local maxima events of joint angular velocity as percentages relative to the entirety of force application for the sword arm elbow as well as the rear hip, knee and ankle.

### Statistical analysis

All statistical tests were performed in SPSS (v.20; IBM, Armonk, NY). Normality was confirmed using a Shapiro-Wilk test, hence data were presented as means ± standard deviation (SD) and between group differences were compared using independent *t*-tests with alpha level set at 0.05. The alpha level was not corrected for multiple comparisons, as attempting to decrease the propensity of a type 1 error can lead to increasing the likelihood of a type 2 error (Perneger 1998). Percentage differences were calculated relevant to the novice group to further demonstrate differences between groups. Rank scoring was assigned according to the temporal sequence of maximal joint angle extension for hip, knee and ankle respectively for each subject and presented as averages for both the skilled and novice groups (e.g. first joint reaching maximal extension scoring 1; second joint scoring 2, last joint scoring 3). A Pearson’s product moment correlation was used to determine the relationship between peak ankle angular velocity, the distal endpoint of the chain, and the discrete kinetic variables of *F*_*Y*_ and Impulse_*Y*_.

## Results

There were no significant differences in movement duration (0.61 ± 0.11 s skilled vs. 0.67 ± 0.17 s novice; *p* = 0.46), yet the skilled group lunged further (1.15 ± 0.11 vs. 0.86 ± 0.16 leg lengths novice; *p* = 0.02). Peak horizontal sword velocity was significantly greater in the skilled group (3.24 ± 0.24 m.s^−1^ skilled vs. 2.69 ± 0.29 m.s^−1^ novice; *p* = 0.02).

There were no significant differences in peak elbow extension velocities (skilled 5.37 ± 1.87 rad·s^−1^ vs. 4.57 ± 1.63 rad·s^−1^ novice; *p* = 0.21), with a large spread of peak elbow extension timing shown with large standard deviations (86 ± 31% of *F*_PushOff_ for novice and 70 ± 28% for skilled). As illustrated in Table 1 there were no significant differences in hip angular velocities between the two groups (*p* = 0.24). The skilled group demonstrated greater peak knee extension velocity, although this was not significant (*p* = 0.17). The skilled group demonstrated significantly greater peak ankle plantarflexion velocity (*p* = 0.02). There was a clear increase in magnitude in a proximal to distal sequence in both groups from hip to ankle.

**Table 1.** Rear leg kinematic variables for novice and skilled groups (mean ± SD)

Joint Kinematics	Novice (*n* = 8)	Skilled (*n* = 7)	% Difference	*p*
Peak hip angle (°)	161 ± 11	159 ± 14	−1.25	0.85
Peak knee angle (°)	165 ± 5	171 ± 6	3.57	0.71
Peak ankle angle (°)	121 ± 12	137 ± 12*	12.40	0.26
Peak hip angular velocity (rad·s^−1^)	2.4 ± 0.9	1.9 ± 0.7	−23.25	0.24
Peak knee angular velocity (rad·s^−1^))	4.6 ± 1.3	6.0 ± 2.4	26.42	0.17
Peak ankle angular velocity (rad·s^−1^)	5.4 ± 2.9	9.1 ± 2.1*	51.03	0.02
Peak hip angular velocity time (%)	84 ± 6	80 ± 17	−4.88	0.45
Peak knee angular velocity time (%)	91 ± 4	88 ± 9	−3.35	0.14
Peak ankle angular velocity time (%)	94 ± 3	94 ± 9	0.00	0.20
Hip rank scoring (target 1)	1.25 ± 0.50	1.00 ± 0.00	–	–
Knee rank scoring (target 2)	2.00 ± 0.80	2.00 ± 0.00	–	–
Ankle rank scoring (target 3)	2.75 ± 0.50	3.00 ± 0.00	–	–

Notes: Temporal sequencing is presented as percentage of push off phase (0% = *F*_*O*,_ 100% = *F*_*TO*)_. Percentage differences are presented relative to novice group results. *T*-test *p* values presented between groups.

*p < 0.05 between novice and skilled.

The skilled group’s average peak extension times occurred earlier than the novice group, starting with hip angular extension velocities occurring at 80% for skilled compared to 84% for novice. Following maximal hip extension there is a general proximal to distal sequence in extension timing for both groups. The rank scoring shows that the skilled group all used a sequential pattern, of 1, 2 and 3 for hip, knee and ankle, whereas this was not the case with the novice group.

**Figure 2.**
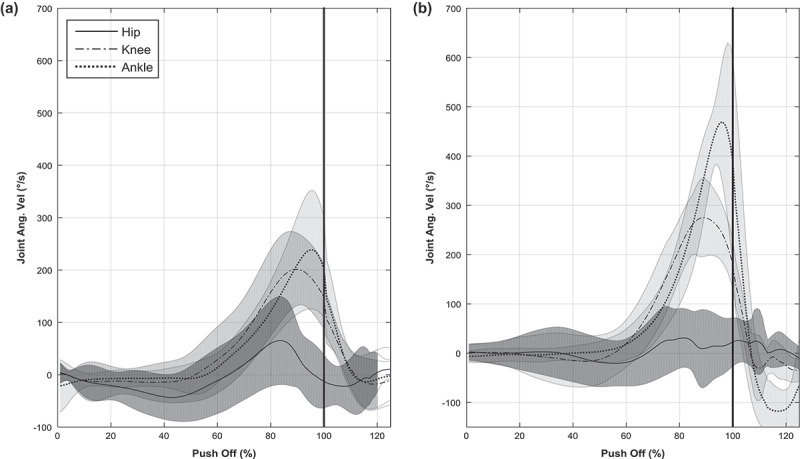
Mean joint angular velocity series data for novice (a) and skilled (b)

Notes: Standard deviations (±1 SD) are plotted either side of each mean, with grey shading depicting the data spread. Extension is positive. Push off phase is from initiation of rear leg force (0%) to take off (100%). The thick vertical line at 100% signifies take off.

Figure 2 visually highlights differences in the rear leg kinematic chain between the novice (2a) and skilled groups (2b). The sequential chain, as quantified in Table 1, can be qualitative presented in this way. The time series of the rear leg angular velocities show a clear sequential kinematic chain in both groups, yet more exaggerated and ending with a significantly greater ankle peak velocity in the skilled group. The sequential extension of the hip, knee and then ankle show an accumulative increase in extension magnitude, with the skilled group ankle plantarflexion seemingly continuing from the point of maximal knee extension.

**Figure 3.**
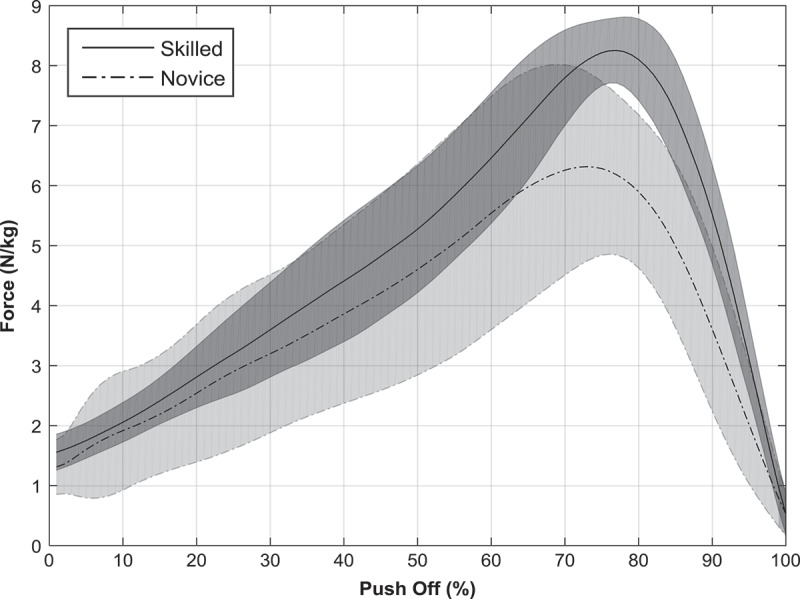
Mean normalized horizontal force data for both novice and skilled groups

Notes: Standard deviations (±1 SD) are plotted either side of each mean, with grey shading depicting the data spread. Push off phase is from initiation of rear leg force (0%) to take off (100%).

There were no significant differences between normalized peak *F*_*Z*_ and normalized Impulse_*Z*_ between both groups. Skilled participants demonstrate significantly greater normalized peak *F*_*Y*_ than novices. The skilled group also demonstrate greater normalized Impulse_*Y*_ than the novice group (Table 2). Figure 3 below displaces normalized horizontal force-time traces for both groups.

**Table 2.** Kinetic variables for novice and skilled groups (mean ± SD)

Kinetic Data	Novice (*n* = 8)	Skilled (*n* = 7)	% Differences	*p*
Peak *F*_*Z*_ (N·kg^−1^)	9.09 ± 2.33	8.54 ± 1.72	−6.24	0.95
Peak *F*_*Y*_ (N·kg^−1^)	6.95 ± 1.92	8.48 ± 0.62*	19.97	0.01*
Application of force time (s)	0.51 ± 0.07	0.54 ± 0.08	5.71	0.40
Impulse_*Z*_ (N·s·kg^−1^)	2.08 ± 0.39	2.08 ± 0.32	0.00	0.93
Impulse_*Y*_ (N·s·kg^−1^)	1.92 ± 0.36	2.51 ± 0.25*	26.64	0.02*

Notes: Percentage differences are presented relative to novice group results. *T*-test *p* values presented between groups.

*p < 0.05 between skilled and novice.

Pearson’s product moment correlation showed a strong positive correlation (*r* = 0.81; *p* = 0.00) between peak ankle angular velocity and *F*_*Y*_ across all participants, yet a weak positive correlation between peak ankle angular velocity and Impulse_*Y*_ (*r* = 0.28; *p* = 0.31).

## Discussion

The results of this study support the initial hypotheses that skilled participants demonstrate clearer proximal to distal sequencing of the rear leg kinematic chain, and greater accumulative angular velocity magnitudes at the hip, knee and ankle joints. The peak rear leg joint angular velocity ranking demonstrates that the skilled fencers use kinematic sequencing to a greater extent than novices. This has been suggested as a more optimal use of a whole limb in propulsive movements (Bobbert and van Soest 2001). With no differences in elbow kinematics between the groups, the significantly greater horizontal sword velocity and lunge distance can be explained through a more effective utilization of the rear leg kinematic chain for greater forward propulsion. This is consistent with previous research demonstrating additional sword velocity is developed through coordination of the lower extremities in the attacking lunge (Yiou and Do 2000; Guilhem et al. 2014). Using a sequential rear leg kinematic chain could possibly be allowing the skilled athletes to better harness a proximal to distal power transfer, as suggested with the increasing angular velocities here, thus developing greater forward propulsion of the system centre of mass (Jacobs et al. 1996; Bobbert and van Soest 2001; Cleather et al. 2015). The assessment of the kinematic chain demonstrated in this paper appears to be an appropriate method to determine athletes effectiveness in using the whole limb in propulsion, and distinguishes between skill level in an applied environment.

The large standard deviations of both groups’ maximal elbow extension velocity timing (±31% skilled vs. ±28% novice) demonstrate individual variability in elbow movement selection. This is perhaps explained with the arm controlling the aim of the sword; therefore, the timing at which the elbow extends may be variable between participants to allow for adjustments so that accuracy can be maintained. According to etiquette in the discipline of fencing with foil, the sword arm must begin to extend prior to movement (Fédération Internationale d’Escrime 2015) for an attack to be deemed valid. Therefore, this limb must always move first, whether this be via shoulder extension\adduction, elbow extension or both.

There was a clear proximal to distal increase of angular velocity magnitudes from the hip to knee in both groups. Descriptively, this was more pronounced in the skilled group (2.4–4.6 rad·s^−1^ for novice hip to knee, compared to 1.9–6.0 rad·s^−1^ skilled), however there were no significant differences between groups when peak joint angular velocities were compared (hip, *p* = 024; knee, *p* = 0.17). The lower hip angular velocity and resulting greater knee angular velocity can best be explained with the skilled fencers using a more advantageous position of the force-velocity curve of muscular contraction (Feltner et al. 1999). Since relatively large force is required to overcome inertial properties of the heavy trunk segment compared to other joints, as well as a large extension and abduction force to move the body, the angular velocity of the hip will be the lowest in both groups. These larger forces would serve as greater input to the power transfer mechanism of the bi-articular rectus femoris (Gregoire et al. 1984; Jacobs et al. 1996; Bobbert and van Soest 2001). It could be postulated that as joint power is a product of net joint moments and angular velocity, and net moment comprised of internal joint forces, the most effective power transfer from this larger muscle via bi-articular design may well be a larger force with the heavier trunk segment inhibiting extension velocity. Although power was not measured in this investigation, this could help to explain the lower, although not significant, hip velocity yet larger knee extension velocity in the skilled group.

A key difference in the skilled group was that this increase in angular velocity magnitude continued distally to the ankle resulting in a significantly greater ankle plantar flexion velocity (9.1 ± 2.1 rad·s^–1^ skilled, vs. 5.4 ± 2.9 rad·s^–1^ novice; *p* = 0.02). The skilled individual ranking averages scored 1.00, 2.00 and 3.00 for the hip, knee and ankle respectively, showing that on an individual level all of the skilled group followed a sequential proximal to distal movement pattern, initiated in the most proximal joint of the rear leg. The novice group rank averages scored 1.25 ± 0.5, 2.00 ± 0.80 and 2.75 ± 0.05 showing that the novice performers did not all follow a set sequential pattern. Only half of the novice group had a clear, proximal-to-distal sequential pattern. This adds weight to theoretical effectiveness of the proximal to distal sequencing evident in the skilled group. Mathematical modelling by Bobbert and van Soest (2001) demonstrated that extending the hip, knee and ankle in a sequential pattern is optimal in explosive jumping movements. In particular, timely extension of the ankle, the smallest and most distal segment with the lowest inertia, is pivotal in achieving maximal jump height. Earlier studies have calculated that 25% of the total work done about the ankle is due to a transfer action from the knee to ankle joint via the gastrocnemius (Bobbert et al. 1986) and optimized with a timely transfer. This supports the findings of this study, with the skilled participants demonstrating better temporal sequencing in a proximal to distal manner. Although the previous findings are predominantly derived from vertical movements, work by Jacobs et al. (1996) has identified similarities in sequential patterning utilizing lower limb biarticular musculature in single leg jumps and the sprint start push off, suggesting that a closed kinematic chain in the lower extremities is transferable to the forward propulsive movement of the rear leg in the fencing lunge investigated in this study.

The timing of mean peak velocity was not statistically different between groups, although there were some noticeable differences in the variability of this timing. The skilled group showed individually greater variation with extension timing standard deviations (±17% hip, ±9% knee and ±9% ankle for skilled, vs. ±6% hip, ±4% knee and ±3% ankle for novice). This highlights that the skilled group had a range of individual extension timing strategies, which warrants further investigation in future research on utilization of the kinematic chain.

The addition of external kinetic data allows for an evaluation of the kinematic sequencing output to a propulsive task. No significant differences were found in normalized vertical kinetic variables. In contrast, significant differences were found in the normalized horizontal kinetic variables with the skilled group demonstrating both greater *F*_*Y*_ and Impulse_*Y*_ than the novice group. This shows that the skilled group not only generated greater force to exploit the impulse-momentum relationship but were also more effective in transferring the rotational movement toward forward propulsion, without expending unnecessary force in the vertical direction. The strong positive correlation between ankle angular plantar-flexion velocity and normalized horizontal peak force (*r* = 0.81; *p* = 0.00) across all participants could suggest that a greater ankle plantar-flexion velocity magnitude, obtained via an effective sequential kinematic chain, results in greater force generation. A strong positive correlation between ankle plantar-flexion velocity and Impulse_*Y*_ would strengthen the notion that an effective kinematic chain results in greater forward propulsion, due to the impulse-momentum relationship, however with a week positive correlation (*r* = 0.28; *p* = 0.31), less effective timing of force application relative to take off may obscure this slightly with some individuals. For example, one novice participant has a large mean ankle plantar-flexion velocity (11.15 rad·s^−1^) which occurs at 101% *F*_PushOff_, which is just after take-off. This pattern is best explained with some individuals achieving greater plantar-flexion velocity once body weight bearing down on the joint is no longer inhibiting the movement. In this regard, high plantar-flexion velocity may actually be a by-product of exerting large peak forces, which as a consequence of the weight reduction occurring at take-off, display strong correlations with the proceeding force.

The primary limitations of this research may be the descriptive study design, along with the small sample size, however it does offer insight into effective kinematic sequencing toward propulsion in a sport specific skill. Limitations can also be associated with multi-site research (e.g. differences in data collection tool specifications). To combat this, collection methodologies were matched as closely as possible, and the same researcher conducted data collection at both sites. Furthermore, although participant numbers at each site were different (site 1, *n* = 9; site 2, *n* = 6), grouping was equally proportioned at both.

Foundations of future research should implement a longitudinal intervention to manipulate the kinematic chain, and assess the changes in performance that arise as a result. Should these differences be identifiable via a simple kinematic chain approach using joint angles, then the provision of feedback on these variables may allow the athlete to subtly alter the kinematic chain and subsequently improve performance.

## Conclusion

These results indicate that more proficient performers in an explosive lunge task use the kinematic chain to a greater extent. This kinematic sequencing facilitates the development of greater angular velocity of the most distal joint, resulting in greater peak horizontal forces and horizontal impulse. This coordination pattern exploits the impulse-momentum relationship resulting in greater forward velocity. The use of joint angles to quantify kinematic sequencing is a useful method to assess whole limb contribution to a propulsive task in an applied setting.
